# Segregated Structure Copolymer of Vinylidene Fluoride and Tetrafluoroethylene Composites Filled with rGO, SWCNTs and Their Mixtures

**DOI:** 10.3390/polym14194105

**Published:** 2022-09-30

**Authors:** Kseniya Shiyanova, Maksim Gudkov, Mikhail Torkunov, Natalia Ryvkina, Igor Chmutin, Galina Goncharuk, Alexander Gulin, Sergey Bazhenov, Valery Melnikov

**Affiliations:** 1 Semenov Federal Research Center for Chemical Physics, Russian Academy of Sciences, 119991 Moscow, Russia; 2 JSC Technopark “Slava”, 117246 Moscow, Russia; 3 Enikolopov Institute of Synthetic Polymeric Materials, Russian Academy of Sciences, 117393 Moscow, Russia

**Keywords:** polymer composites, segregated structure, reduced graphene oxide, single-walled carbon nanotubes, electrical conductivity, mechanical properties, absorption of microwave radiation

## Abstract

This work is devoted to the formation and study of polymer composites with a segregated structure filled with single-walled carbon nanotubes (SWCNTs), reduced graphene oxide (rGO), and their mixtures. For the first time, polymer composites with a segregated structure filled with rGO/SWCNTs mixtures were obtained. A copolymer of vinylidene fluoride and tetrafluoroethylene (P(VDF-TFE)) was used as a polymer matrix. At a fixed value of the total mass fraction of carbon nanofillers (0.5, 1, and 1.5 wt%), the rGO/SWCNTs ratio was varied. The composites were examined using scanning electron microscopy, wide-range dielectric spectroscopy, and tested for the compression. The effect of the rGO/SWCNTs ratio on the electrical conductivity and mechanical properties of the composites was evaluated. It was shown that, with a decrease in the rGO/SWCNTs ratio, the electrical conductivity increased and reached the maximum at the 1 wt% filling, regardless of the samples’ composition. The maximum value of electrical conductivity from the entire data set was 12.2 S/m. The maximum of elastic modulus was 378.7 ± 3.5 MPa for the sample with 1 wt% SWCNTs, which is 14% higher than the P(VDF-TFE) elastic modulus. The composite filled with a mixture of 0.5 wt% rGO and 0.5 wt% SWCNTs reflected 70% of the electromagnetic wave energy from the front boundary, which is 14% and 50% more than for composites with 1 wt% SWCNTs and with 1 wt% rGO, respectively. The lowest transmission coefficient of ultra-high frequencies waves was obtained for a composite sample with a mixture of 0.5 wt% rGO and 0.5 wt% SWCNTs and amounted to less than 1% for a 2 mm thickness sample.

## 1. Introduction

The last two decades have seen an increase in the number of works devoted to the use of carbon nanomaterials. The discovery of fullerenes (C60) in 1985 [1], which was awarded the Nobel Prize, created an entirely new branch of carbon chemistry and aroused significant research interest in its new forms, and subsequent discoveries of carbon nanotubes (CNTs) and graphene further expanded this area [2]. Carbon nanomaterials have unique properties, including a high mechanical strength, excellent electrical and thermal conductivity, high specific surface area, etc. These characteristics justify great prospects in using carbon nanomaterials to create electrically conductive polymer composites (EPCs) [3].

The development of electrically conductive composite materials is an important task in materials science. This is due to the active growth of the demand for flexible electronics, radio frequency identification (RFID) tags, flexible sensors [4] and materials for absorbing electromagnetic radiation [5].

Currently, many works have been published on the development and study of EPCs [3,6,7,8,9,10]. The optimal distribution of electrically conductive nanosized fillers in a polymer matrix is still one of the main problems [11] preventing wide use of such materials in industry. To achieve high values of electrical conductivity, a high concentration of an electrically conductive filler is required, which leads not only to a fundamental change in the properties of the material, but also to an increase in the cost of the final composite.

A significant reduction in the amount of the filler required for the demanded level of electrical conductivity of the material can be achieved by forming a segregated structure [12], in which the conductive phase is deliberately distributed unevenly and localized at the boundary between the polymer particles. Such a distribution leads to an increase in the local concentration of the filler compared to the uniform distribution that is traditionally realized by mechanical mixing of the components [6]. The electrical conductivity of EPCs with a segregated structure is recorded already at a content of the conductive filler of less than 0.1 wt%, which is significantly lower than the percolation threshold for composites with a uniform distribution of particles, which is usually 1–10 wt%. Such an approach for obtaining composite materials is promising, since it makes it possible to achieve high values of electrical conductivity at a low content of the electrically conductive filler [13].

Grunlan et al. [14] are the founders of electrically conductive composites with a segregated network structure. In their work, SWCNTs were stabilized in a dispersion with polyvinyl acetate, and then films were made from the suspension. The maximum electrical conductivity was achieved at a loading of 4 wt% and was approximately 10 S/m. Composites with the formation of a segregated structure based on rGO have also been studied in detail [6,13,15]. For example, graphene oxide (GO) and a polymer powder were dispersed in a water–alcohol solution under an ultrasonic treatment to disperse graphene. GO was reduced to rGO with hydrazine. Ultra-high molecular weight polyethylene (UHMWPE) particles coated with rGO were subsequently pressed at 200 °C to perform the morphological observations and electrical conductivity measurements. As expected, a polymer composite with rGO and UHMWPE was successfully obtained. The electrical conductivity at 0.6 vol% rGO reached 10^−1^ S/m [16]. In [17], the authors used preliminarily prepared rGO, which was dispersed in an alcohol with an ultrasonic treatment, and the solvent was evaporated in the presence of a polymer powder. Composites based on UHMWPE, multi-walled carbon nanotubes (MWCNTs), and GO were prepared from a water–alcohol dispersion with further pressing at room temperature. The electrical conductivity was 10^−5^ S/m for composites with GO, and 10^−2^ S/m for composites with MWCNTs.

In [18], EPCs were prepared based on SWCNTs/polystyrene and SWCNTs/polycarbonate. SWCNTs were dispersed in chloroform with polyphenyleneethynylene. The authors used the method of distributing the filler in a polymer suspension in an alcohol and obtained the electrical conductivity equal to 6.89 S/m at a load of 7 wt% SWCNTs for composites based on polystyrene (which is 14 orders of magnitude higher than that of pure polystyrene) and 4.81×10^2^ S/m for composites based on polycarbonate at the same load. The method developed in [18] is applicable to various initial polymers and does not require prolonged sonication. Using the Raman spectra, the authors of [19] showed that the preliminary thermal annealing of SWCNTs significantly improved their distribution in polystyrene, which contributed to higher values of electrical conductivity.

A number of papers present results showing the advantages of using mixtures of carbon nanotubes with graphene as fillers for various polymer composites [20,21,22]. The presence of one-dimensional and two-dimensional materials contributes to the manifestation of a synergistic effect, which leads to an improvement in electrophysical, mechanical and electromagnetic shielding efficiency properties. The composites studied in the literature are systems obtained either by mixing in a melt or by mixing solutions. However, we were unable to identify any work that would consider the synergistic effect of a mixture of carbon nanotubes with graphene in polymer composites of a segregated structure.

In this work, composites of a segregated structure based on P(VDF–TFE) filled with rGO, SWCNTs, and their mixtures were obtained and studied. The effect of SWCNTs and a rGO/SWCNTs mixture on the electrical conductivity and mechanical properties of EPCs with a segregated structure was studied for the first time. An analysis of the coating of carbon nanomaterials on the surface of polymer particles was carried out, and the morphology of brittle fractures and thin sections of the composites was also studied. A possibility of using EPCs as a shield of electromagnetic interference in the microwave range was considered. It was shown that the best electromagnetic interference parameters were demonstrated by a composite containing a mixture of rGO and SWCNTs, which is due to the synergistic effect.

## 2. Materials and Methods

### 2.1. Materials

A copolymer of (P(VDF-TFE) (trademark F-42V GOST 25428-82, LLC HaloPolymer Kirovo-Chepetsk, Russia) was used as a polymer matrix in the study. Isopropyl alcohol (99.8%, LLC Ruskhim.ru, Russia) was used as a solvent.

To form an electrically conductive network, an aqueous suspension of graphene oxide (GO, C = 6.9 mg/mL) was used, which was provided by Graphene Technologies Russia (www.graphtechrus.com, Russia). To transfer GO to the reduced form, hydrazine hydrate 100% (LLC Ruskhim.ru, Russia) was used. A detailed characterization of this GO sample is presented in our previous work [6].

SWCNTs from OCSiAl were utilized (https://ocsial.com, Russia). According to the product data sheet, there was a number-based particle size distribution: d10 1.2–1.45 nm, d50 1.6–1.8 nm, d90 1.9-2.2 nm, G/D range > 90 (Raman at 532 nm), a length-to-diameter ratio of 10^3^–10^4^, 1/3 metal and 2/3 semiconductor carbon tubes. SWCNTs were preliminarily cleaned from residual catalyst particles. To do this, the required sample of SWCNTs was placed in a 5M HCl solution, and the dispersion was stirred for 1 day with a magnetic stirrer. Then, the mixture was filtered on a Schott filter and washed with distilled water until pH 6. The resulting SWCNTs were dried in an oven at 70 °C in air for 12 h. 

### 2.2. Composite Preparation Method

For the study, samples of EPCs with a segregated structure based on P(VDF-TFE) were made. 0.5, 1, and 1.5 wt% of rGO, SWCNTs or their mixture were introduced into composite samples. At each fixed value of the fillers weight fraction (ω) in composites with a mixture of fillers, the rGO/SWCNTs ratio was varied. The total concentrations of the nanofillers were chosen based on our previous study results [6]. It has been shown that in the rGO case, the optimal filler concentration in order to achieve high electrical conductivity and well mechanical properties is 1 wt%. Next, 0.5 and 1.5 wt% concentrations were chosen to test the hypothesis of 1 wt% optimum in the case of rGO/SWCNT mixtures. The compositions of the samples are shown in Table 1. It should be noted that all selected filler concentrations were above the percolation threshold for such systems [13,15,16].

The preparation of EPCs with a segregated structure can be divided into several stages. At the first stage, it was required to form a homogeneous dispersion of carbon fillers. The required amount of GO, SWCNTs, or their mixtures in a given ratio was placed in isopropyl alcohol (at a ratio of 20 mg of a carbon filler or their mixture per 100 mL of solvent). This mixture, cooled in an ice bath, was sonicated with a power of 400 W for maximum disaggregation of the components for 40 min (4 times for 10 min with breaks of 5 min). At the last step of ultrasonic treatment, a P(VDF-TFE) powder was added to the mixture to disaggregate polymer particles. The second stage was followed by the removal of the liquid phase. First the mixture was dried to form a slightly wet powder on a rotary evaporator at reduced pressure, formed with a water jet pump, and 40 °C. Then, the wet powder coated with carbon nanoparticles was dried completely in an oven at 80 °C for 12 h. If GO was present in the sample composition, the coated dry powder was treated with hydrazine vapor for 12 h at 80 °C to convert GO into a reduced electrically conductive form (rGO). The last stage included pressing of the obtained coated polymer particles at 200 °C and a pressure of 140 kg/cm^2^ to form a composite with a single electrically conductive network inside.

### 2.3. Sample Analysis Methods

The analysis of the surface morphology of the polymer powder particles with an electrically conductive coating, as well as of the brittle fractures of the composites, was performed using a Prisma E scanning electron microscope (Thermo Scientific, Brno, Czech Republic). The samples were placed on a carbon tape and sputtered with a 10 nm thick layer of gold (Q150R ES, Quorum Technologies, Laughton, UK) for the better charge drain and then fixed on an L-shaped holder perpendicular to the optical axis of the microscope. The analysis was carried out in a high vacuum mode at an accelerating voltage of 2–5 kV. To create a brittle fracture, a sample, in the form of a disk with the diameter of 12 mm and 2 mm thick, was preliminarily placed in liquid nitrogen for 30 min.

To study the electrical conductivity of the composites in the frequency range of 20 Hz–5 MHz, disks with the diameter of 12 mm and 2 mm thick were prepared. The electrical characteristics were measured using an LCR-78105G wide-range dielectric spectrometer (GW Instek, New Taipei City, Taiwan). For testing, the electrodes were applied to the flat ends of the sample, which were covered with a thin layer of electrically conductive glue (Keller, Saint Petersburg, Russia).

The permittivity of the studied composites in the microwave frequency range of the electromagnetic field was measured by the resonator method in a rectangular resonator using panoramic R2 series meters (JSC NPK MERA, Krasnodar, Russia). The samples had a shape of a rectangular parallelepiped with the dimensions of 50 mm × 1 mm × 0.5 mm.

To obtain the physical and mechanical characteristics, mechanical compression tests of the composite samples were carried out using an Autograph AGS-10 kNG universal testing machine (Shimadzu, Kyoto, Japan). For the study, cylinders with a diameter of 5 mm and a height of 10 mm were made. The compression was carried out at room temperature at a rate of 2 mm/min.

## 3. Results and Discussion

### 3.1. Surface Morphology of Coated Polymer Particles and Brittle Cleavages of Composite

**Figure 1:**
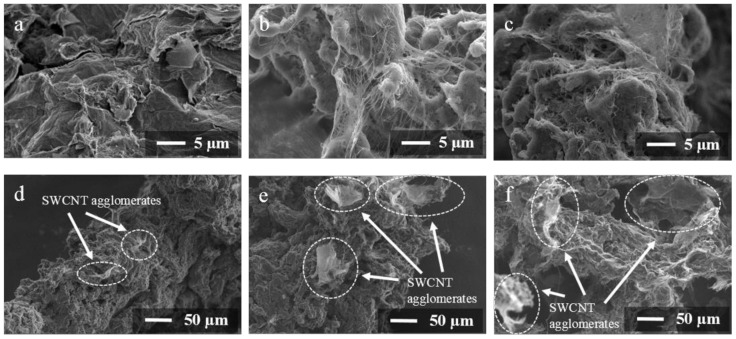
SEM images of the polymer powder particles coated with 1 wt% carbon nanomaterials: (**a**) 1FPC-0, (**b**) 1FPC-50 and (**c**) 1FPC-100. SEM images of the polymer powder particles coated with (**d**) 1, (**e**) 1.25 and (**f**) 1.5 wt% SWCNTs.

Figure 1 shows SEM images of the polymer powder particles coated with 1 wt% carbon fillers. For the 1FPC-0 sample containing only rGO as a filler, graphene sheets are clearly visible in the SEM images (Figure 1a). Polymer particles coated with rGO and SWCNTs in a ratio of 1:1 (1FPC-50) and exclusively SWCNTs (1FPC-100) (Figure 1b and Figure 1c, respectively) have a fairly uniform distribution of the filler over the surface of polymer particles, which indicates a homogeneous distribution of carbon fillers in the dispersion due to its preliminary ultrasonic treatment.

However, it has been found that, when exclusively SWCNTs are applied to the polymer powder particles, agglomerates are formed, and their size increases with an increase in the mass fraction of SWCNTs in a sample (Figure 1d–f). For the 1FPC-100 sample containing 1 wt% SWCNTs, the size of agglomerates does not exceed 50 μm (Figure 1d), while the polymer particles coated with 1.5 wt% SWCNTs (1.5FPC-100) have 100 µm-sized agglomerates on their surface (Figure 1f).

**Figure 2:**
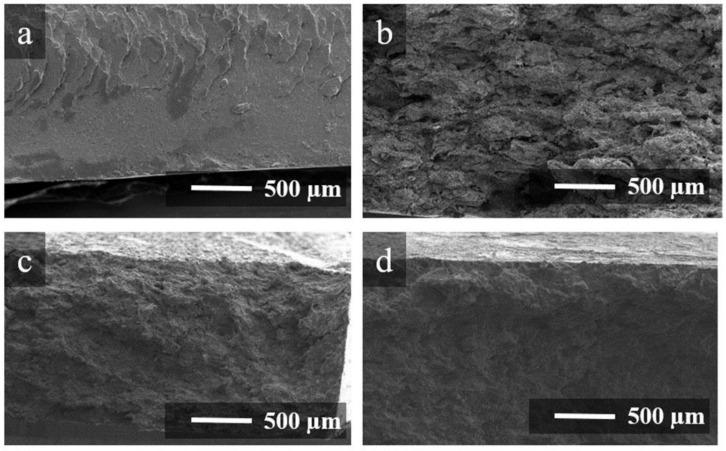
SEM images of brittle fractures of pure (P(VDF-TFE)) (**a**), 1FPC-0 (**b**), 1FPC-50 (**c**), and 1FPC-100 (**d**).

Figure 2 shows SEM images of the brittle fractures of 1FPC-0, 1FPC-50 and 1FPC-100 composites, as well as of pure P(VDF-TFE) after hot pressing. A fairly smooth texture is observed for the brittle fracture of the pure polymer. Sample 1FPC-0 (Figure 2b), which contains only rGO as a filler, has a better developed loose texture than the pure polymer fracture. Additionally, samples 1FPC-50 and 1FPC-100 have a similar structure. Optical images of thin sections from composite samples after pressing are shown in Figure S1. It can be seen from the presented images that the composites structure is indeed segregated, and the filler forms conductive boundaries separating the polymer domains.

### 3.2. Electrical Conductivity

**Figure 3:**
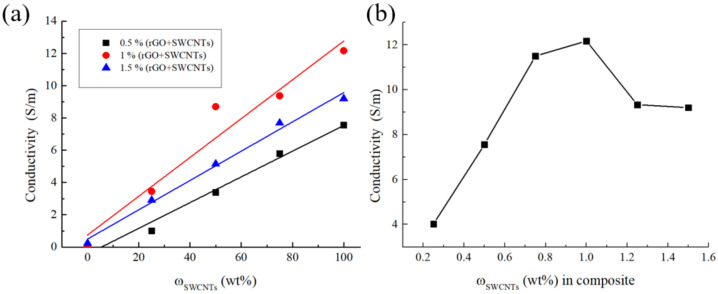
Dependence of the electrical conductivity on the mass content of SWCNTs in a mixture of carbon fillers (**a**), and dependence of the electrical conductivity on the mass content of SWCNTs in the composite (**b**).

To study the electrical properties of the composites, the frequency dependences of the electrical conductivity were obtained (Figure S2). As part of the work, the dependence of the electrical conductivity on the mass content of SWCNTs in a mixture of carbon fillers was studied (Figure 3a). It was shown that the electrical conductivity increased with an increase in the proportion of SWCNTs in the mixture of carbon fillers at any total concentration of carbon fillers. This is due to the higher electrical conductivity of SWCNTs compared to rGO. The literature sources give rather different electrical conductivity values for rGO and SWCNTs. However, we can say with confidence that the values differ by at least an order of magnitude. For rGO reduced with hydrazine hydrate, the electrical conductivity is ≈ 10^3^ S/m [23], and for SWCNTs, it is ≈ 10^4^ S/m [24]. 

The data in Figure 3a demonstrate that the dependence of the electrical conductivity on the total mass fraction of carbon fillers has an extremum. To refine the obtained data, additional samples filled exclusively with SWCNTs with different fillings were made (Figure 3b). An analysis of the electrical conductivity showed that the dependence was indeed extreme, and the maximum was in the region of 1 wt% SWCNTs. The electrical conductivity value at the maximum is 12.16 ± 0.001 S/m. It should be noted that the value obtained has a good correlation with the literature data on composites of a segregated structure filled with CNT (Table 2). However, we were unable to find any publications on electrically conductive composites of a segregated structure filled with SWCNTs in the pure form or as a component of a mixture, which emphasizes the importance of our study. It should be noted that all the SWCNTs concentrations we studied are significantly above the percolation threshold. 

The atypical behavior of the concentration dependence of the composite’s electrical conductivity is related to the fact that, at high SWCNT concentrations, the number of agglomerates increases, due to the complexity of the coating organization on the surface of polymer particles (Figure 1d–f). With an increase in the size of SWCNT agglomerates, the probability of a local decrease in the thickness of the coating in some places increases, which leads to the formation of zones with increased resistance, and, as a result, to a decrease in the integral value of the electrical conductivity of the composite. Additionally, one of the reasons for the decrease in the electrical conductivity in samples with the SWCNTs content > 1 wt% is a probable increase in the porosity of the carbon coating on the surface of polymer particles. The increased porosity leads to a reduced volume concentration of the filler. The assumption about the porosity increase was confirmed by the direct measurement of the density by the hydrostatic weighing method (Figure S3). It has been shown that the experimentally determined density of composites decreases with an increase in the mass fraction of SWCNTs, which is explained by an increase in the volume fraction of pores.

### 3.3. Mechanical Properties of Electrically Conductive Composites

The resulting composites were tested for compression. Typical tress σ–strain ε curves are shown in Figure S4. The stress–strain curves of composites are similar to that of unfilled polymer. Thus, the effect of fillers on mechanical properties of composites is not too significant. The obtained mechanical characteristics are given in Table S1.

**Figure 4:**
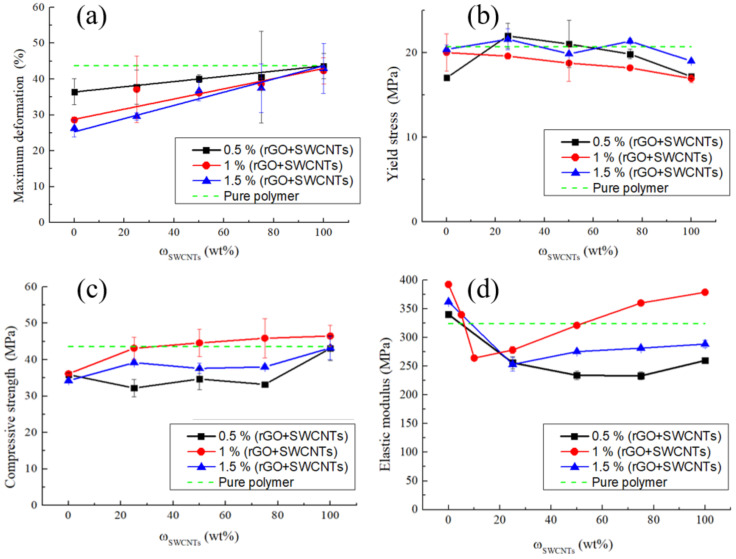
(**a**) Maximum deformation, (**b**) yield stress, (**c**) compressive strength, and (**d**) elastic modulus of obtained composites plotted against the mass content of SWCNTs in the rGO/SWCNTs mixture.

Figure 4a shows the dependences of the deformation at the point of maximum stress on the mass fraction of SWCNTs in a mixture of carbon fillers in the composite. The introduction of rGO without SWCNTs into the polymer matrix reduces the deformation at the ultimate strength compared to the pure polymer, and also leads to a decrease in the maximum deformation. The contact surfaces of polymer particles coated with rGO represent low shear planes, that leads to a decrease in the strength compared to the pure polymer. As a result, the deformation also decreases. It is also worth noting that, in samples without SWCNTs, an increase in the rGO content leads to a decrease in the deformation. 

When rGO is replaced by SWCNTs, the deformation of composites increases monotonically at all the filler concentrations. Regardless of the total mass fraction of filling (0.5, 1, 1.5 wt%), the maximum relative strains are achieved in the composites filled exclusively with SWCNTs and amount to 43.6, 42.3, and 43.0%, respectively, which, considering the data scatter, are equal to the deformation of the pure polymer (44%).

At the filler concentrations of 1 wt% and 1.5 wt%, there is a slight decrease in the yield stress with an increase of ω_SWCNTs_ in the mixture of fillers (Figure 4b). The yield stress was determined at the point where the stress declines from the linear Hookean behavior. The dependence for the composites containing 0.5 wt% carbon fillers is similar, but an outlier can be noted for the samples without SWCNTs. This outlier is within the scatter of the data.

Figure 4c shows the dependence of the composites’ compressive strength on the mass fraction of SWCNTs in a mixture of carbon fillers. In samples containing only rGO, there is a decrease in the strength by 22% compared to the pure polymer. P(VDF-TFE)) is a plastic polymer and the rGO coating is a low shear plane. This leads to a decrease in the strength.

Replacing rGO with nanotubes causes a monotonic increase in the compressive strength. This growth is most noticeable at a carbon filler concentration of 1 wt%. In this case, the strength is 46.5 ± 2.9 MPa, which exceeds that of the pure polymer.

The dependence of the compressive strength for samples with a mass fraction of filling of 0.5 wt% is not similar to the others. At ω_SWCNTs_ from 0 to 75%, the strength of the composite is constant, but when the polymer is filled with only SWCNTs, the strength of the composite increases to 43.1 ± 3.4 MPa, which corresponds to the compressive strength of the pure polymer.

Summarizing the above data, we can draw the following conclusion. The structural roles of rGO and SWCNTs in the composites are different. The influence of graphene on the mechanical behavior of P(VDF-TFE)) was investigated in [6]. Increasing the SWCNTs concentration enhances the reinforcement effect. Apparently, at the SWCNTs concentration of 1.5 wt%, the porosity and inhomogeneity of the distribution of nanotubes has an adverse effect (Figure 1d–f and Figure S1), and therefore, the filler concentration of 1 wt% is optimal.

The introduction of rGO into the polymer matrix also leads to a slight increase in the elastic modulus compared to the pure polymer (Figure 4d). For the composite containing 1 wt% rGO, the modulus has the highest value of 390 MPa. The composites containing 0.5 and 1.5 wt% rGO have an elastic modulus lower than that of sample 1FPC-0. Thus, a maximum of the elastic modulus is observed depending on the mass fraction of rGO in the mixture of fillers.

When SWCNTs are added to rGO (ω_SWCNTs_ = 25 wt%), a decrease in the elastic modulus is observed at all the mass contents of carbon fillers. To check the correctness of the data, we made additional samples containing a 1 wt% carbon filler, with a ratio of fillers 5:95 and 10:90. Even at a SWCNTs to rGO ratio of 5:95, the elastic modulus decreased, but the minimum was reached at ω_SWCNTs_ = 10 wt%. Further, with an increase in the SWCNTs content, the elastic modulus begins to increase and reaches its maximum for samples containing only SWCNTs. The highest value of the modulus is achieved for the composite filled with 1 wt% SWCNTs (1FPC-100). The modulus is 378.7 ± 3.5 MPa, which is 14% higher than the elastic modulus of the pure polymer.

### 3.4. Reflection, Absorption and Transmission Coefficients in the Microwave Range

**Figure 5:**
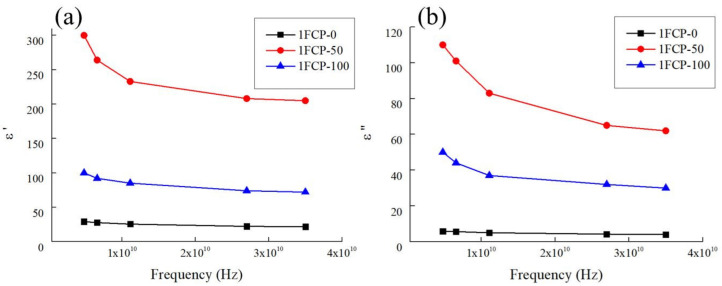
Experimental frequency dependences of the real (**a**) and imaginary (**b**) parts of the complex permittivity for the samples 1FPC-0 containing only 1 wt% rGO, 1FPC-50 containing a mixture of 0.5 wt% SWCNTs and 0.5 wt% rGO, 1FPC-100 containing only 1 wt% SWCNTs.

For the 1FPC-100 containing only 1 wt% SWCNTs, 1FPC-50 containing a mixture of 0.5 wt% SWCNTs and 0.5 wt% rGO, 1FPC-0 containing only 1 wt% rGO, the dependences of the real (ε’) and imaginary (ε”) parts of the complex permittivity on the frequency of the electric field in the microwave frequency range were measured. The measurement results are shown in Figure 5.

Based on these experimental values of the dielectric properties, the coefficients of reflection from the leading edge (R), absorption (A), and transmission (T) of ultra-high frequencies (UHF) waves for flat samples with a thickness (h) from 1 to 5 mm were calculated. The calculations were carried out using Formulas (S1)–(S6), which are based on the Fresnel equations [32].

About 70% of the electromagnetic wave energy is reflected from the front boundary of the composite material sample for 1FPC-50, about 60% and 35% for 1FPC-100 and 1FPC-0, respectively. Moreover, the reflection coefficient does not depend on the sample thickness. The energy transmission coefficient of an electromagnetic wave already significantly depends on type and thickness of the composite material sample.

**Figure 6:**
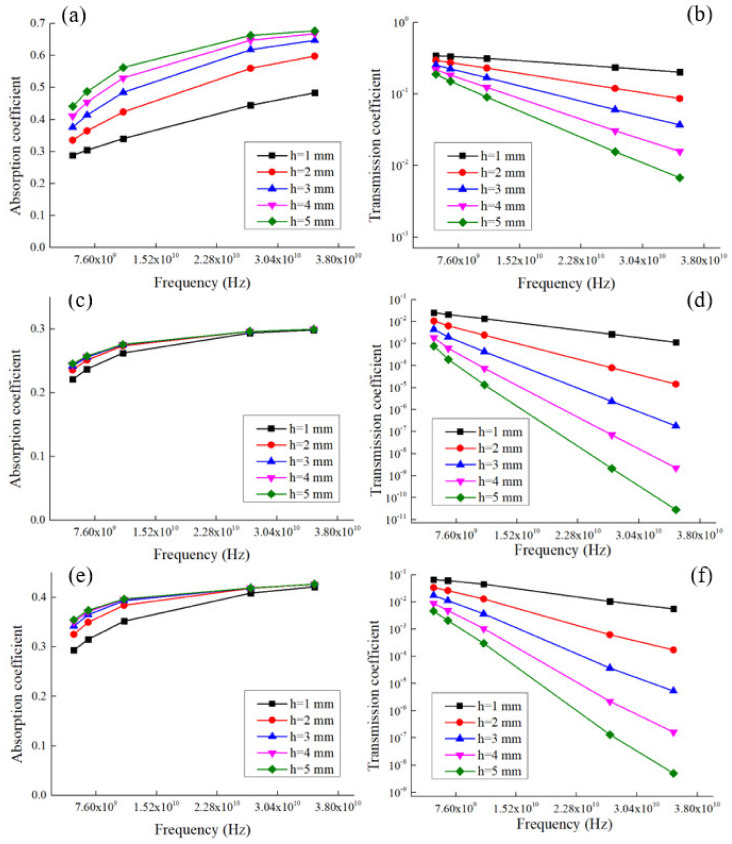
Frequency dependence of absorption coefficient (**a**,**c**,**e**) and transmission coefficient (**b**,**d**,**f**) for 1FPC-0, 1FPC-50 and 1FCP-100 samples, respectively.

Figure 6 shows the dependence of the transmission coefficient on the frequency of the wave. It should be note that the lowest transmission coefficient of UHF waves was obtained for a composite sample 1FPC-50. It was less than 1% for a sample thickness of 2 mm. With increasing thickness, T decreases. These results demonstrated the presence of a synergistic effect of one-dimensional and two-dimensional filler in a mixture for polymer composites with a segregated structure. The nature of this effect needs to be studied in detail in the future, as well as to form the theoretical foundations for its occurrence in segregated structure composites.

Materials with this composition can be used as shields for electromagnetic waves in the UHF range. Such shields can serve to protect electronic equipment and people from the effects of microwave radiation from cellular base stations, radars near airports, etc.

## 4. Conclusions

In this work, for the first time, we obtained electrically conductive polymer composites with a segregated structure using rGO/SWCNTs mixtures in various ratios as fillers. The SEM analysis of the powders coated with a mixture of carbon nanomaterials before pressing showed that the coating was distributed fairly evenly over the polymer particles’ surface. However, in the powder samples coated with only SWCNTs, agglomerates were observed in the coating, with the size of the detected agglomerates increasing with an increase in the concentration of nanotubes.

An almost linear electrical conductivity dependence on the content of rGO, SWCNTs, and the rGO/SWCNTs mixture on the polymer particles was shown. For the 1FCP-100 sample containing only 1 wt% SWCNTs, the electrical conductivity maximum of 12 S/m was reached. It was found that for all the composites, regardless of their composition, the electrical conductivity dependence on the mass fraction of the filler had an extremum in the region of ~1 wt%.

When studying the mechanical properties, it was noted that an increase in the proportion of SWCNTs in the composites resulted in an increase in the maximum deformation. In turn, the elastic modulus increased with the addition of rGO to the polymer, and then decreased with the addition of SWCNTs to rGO and the formation of their mixture. Moreover, the larger the share of SWCNTs in the SWCNTs/rGO mixture, the greater the values of the elastic modulus. Replacing rGO with nanotubes causes a monotonic increase in the compressive strength. This growth is most noticeable at a carbon filler concentration of 1 wt%. In this case, the strength is 46.5 ± 2.9 MPa.

It was found that about 70% of the electromagnetic wave energy is reflected from the front boundary of the composite material sample for 1FPK-50, about 60% and 35% for 1FPK-100 and 1FPK-0, respectively. The lowest transmission coefficient of UHF waves was obtained for a composite sample with a mixture of 0.5% rGO and 0.5% SWCNTs. For a sample with a thickness of 2 mm, it was less than 1%, which indicates the synergistic effect of the rGO/SWCNTs mixture in composites with a segregated structure, and is shown for the first time. The data indicate that the obtained materials are promising for use as electromagnetic interference shielding in the microwave range.

## Figures and Tables

**Table 1:** Compositions of fabricated samples of electrically conductive composite materials.

Sample	ω_rGO+SWCNTs_, %	ω_SWCNTs_ in a Mixture of Carbon Fillers, wt%	ω_rGO_ in a Mixture of Carbon Fillers, wt%
0.5FPC-0	0.5	0	100
0.5FPC-25	0.5	25	75
0.5FPC-50	0.5	50	50
0.5FPC-75	0.5	75	25
0.5FPC-100	0.5	100	0
1FPC-0	1	0	100
1FPC-25	1	25	75
1FPC-50	1	50	50
1FPC-75	1	75	25
1FPC-100	1	100	0
1.5FPC-0	1.5	0	100
1.5FPC-25	1.5	25	75
1.5FPC-50	1.5	50	50
1.5FPC-75	1.5	75	25
1.5FPC-100	1.5	100	0

**Table 2:** Experimental values of the electrical conductivity of polymer composites based on CNT described in the literature.

Matrix	Filler	Electrical Conductivity, S/m	References
Poly(phenylene ethynylene)/ polycarbonate composite	SWCNTs, 7 wt%	4.81 × 10^2^	[17]
Polystyrene	SWCNTs, 2 wt%	10^−2^	[18]
Polylactic acid	MWCNTs, 2 wt%	20	[25]
Ultra-high molecular weight polyethylene	MWCNTs, 2 wt%	2.1	[26]
Epoxy vitrimer	MWCNTs, 4 wt%	6.8	[27]
Polypropylene	MWCNTs, 1 wt%	14.2	[28]
Epoxy resin	MWCNTs, 1 wt%	34.2	[29]
Polyvinylidene fluoride	MWCNTs, 4 wt%	74.5	[30]
L,d-poly(lactic acid)/ 4-pentyl-4-biphenylcarbonitrile	SWCNTs, 0.05 wt%	0.024	[31]
P(VDF-TFE)	SWCNTs, 1 wt%	12.16	This article

## Data Availability

Non-applicable.

## Supplementary Materials

The following supporting information can be downloaded at: https://www.mdpi.com/article/10.3390/polym14194105/s1, Figure S1: Optical images of thin sections of composite samples (a) 1FPC-0, (b) 1FPC-50 and (c) 1FPC-100; Figure S2: Frequency dependence of the electrical conductivity of composites 1FPC-0, 1FPC-50 and 1FPC-100; Figure S3: Dependence of the composites density on the SWCNTs weight content in the composite. The experimental density of the samples was calculated using hydrostatic weighing in heptane and on the air. The theoretical density was calculated on the basis of the theoretical density of the P(VDF-TFE) 1.9 g/cm^3^ and SWCNT – 2 g/cm^3^.; Figure S4: Typical compressive stress σ–strain ε curves for pure P(VDF-TFE) and composites filled by 1% by weight of rGO, SWCNT or their mixtures; Table S1: Main mechanical characteristics of the resulting composites; Formulas S1–S6: Calculation of reflection, transmission and absorption coefficients.
