# Supplementary material for: Segregated Structure Copolymer of Vinylidene Fluoride and Tetrafluoroethylene Composites Filled with rGO, SWCNTs and Their Mixtures
Source: Polymers (Basel). 2022 Sep 30;14(19):4105. doi: 10.3390/polym14194105 (PMC9570613; doi:10.3390/polym14194105)
Supplement: Supplementary file 1 [file polymers-14-04105-s001.zip › polymers-1899388-supplementary.pdf]

Segregated structure copolymer of vinylidene fluoride and tetrafluoroethylene composites filled with rGO, SWCNTs and their mixtures

Kseniya Shiyanova[1], Maksim Gudkov[1], Mikhail Torkunov[1], Natalia Ryvkina[1], Igor Chmutin[2], Galina Goncharuk[3], Alexander Gulin[1], Sergey Bazhenov[1], Valery Melnikov[1,*]

[1] Semenov Federal Research Center for Chemical Physics, Russian Academy of Sciences, 119991 Moscow, Russia
[2] JSC Technopark "Slava", 117246 Moscow, Russia
[3] Enikolopov Institute of Synthetic Polymeric Materials, Russian Academy of Sciences 117393 Moscow, Russia
[*] Correspondence: mvpchph@yandex.ru

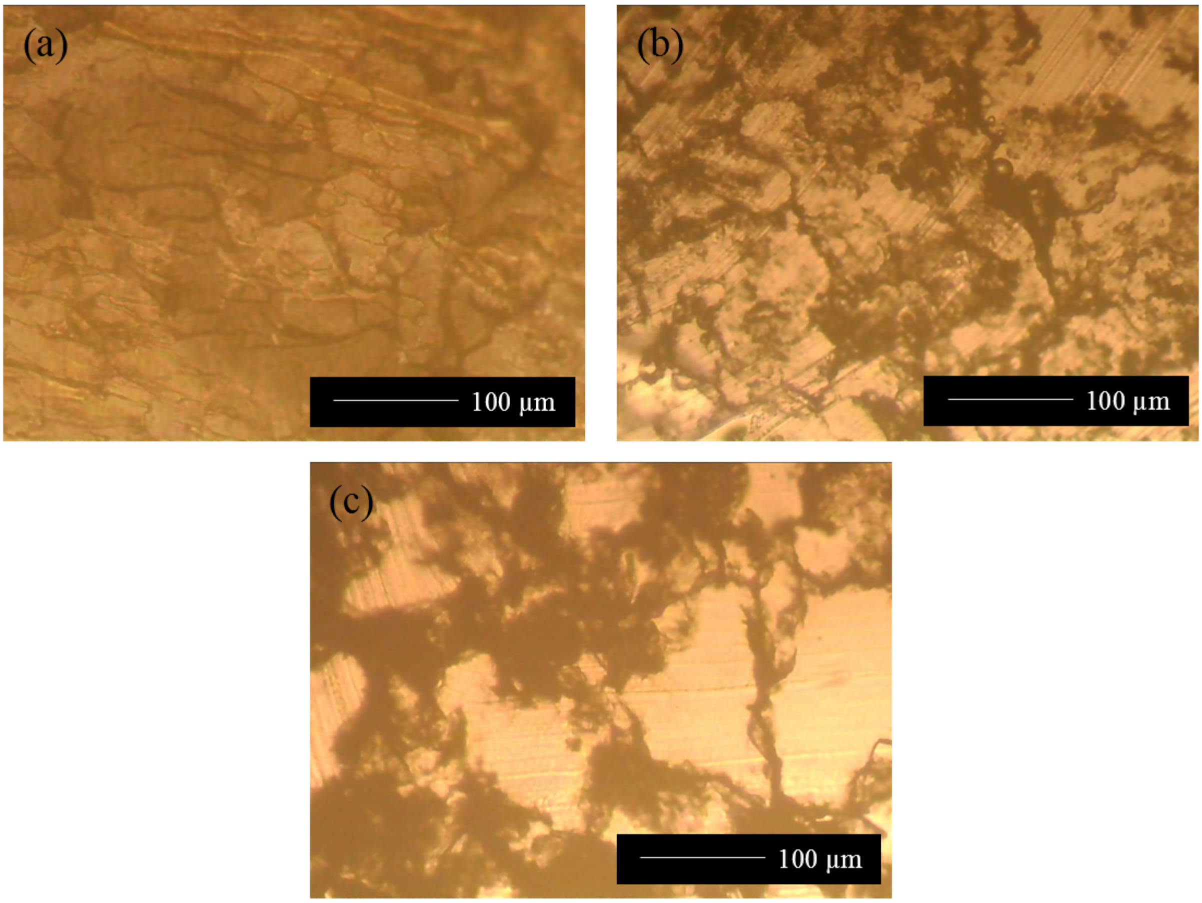

Figure S1. Optical images of thin sections of composite samples (a) 1FPC-0, (b) 1FPC-50 and (c) 1FPC-100.

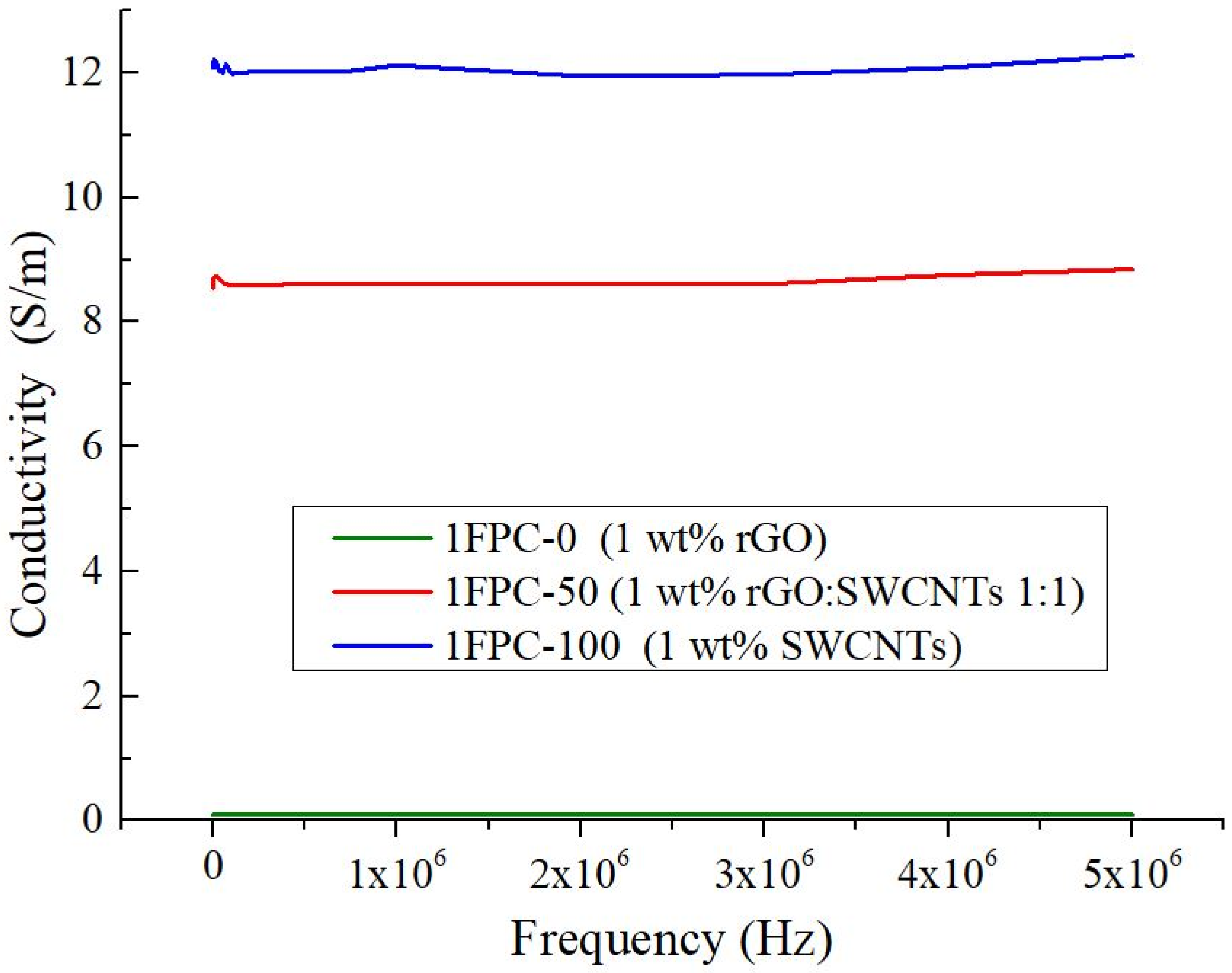

Figure S2. Frequency dependence of the electrical conductivity of composites 1FPC-0, 1FPC-50 and 1FPC-100.

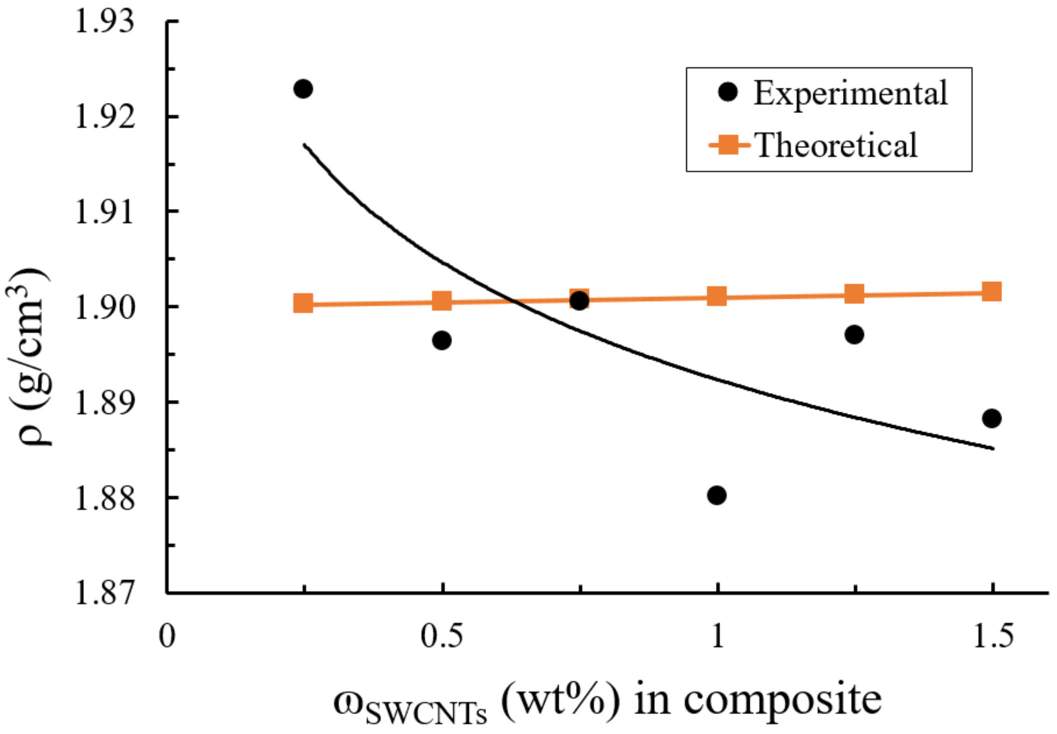

Figure S3. Dependence of the composites density on the SWCNTs weight content in the composite. The experimental density of the samples was calculated using hydrostatic weighing in heptane and on the air. The theoretical density was calculated on the basis of the theoretical density of the P(VDF-TFE) 1.9 g/cm^3 and SWCNT – 2 g/cm^3.

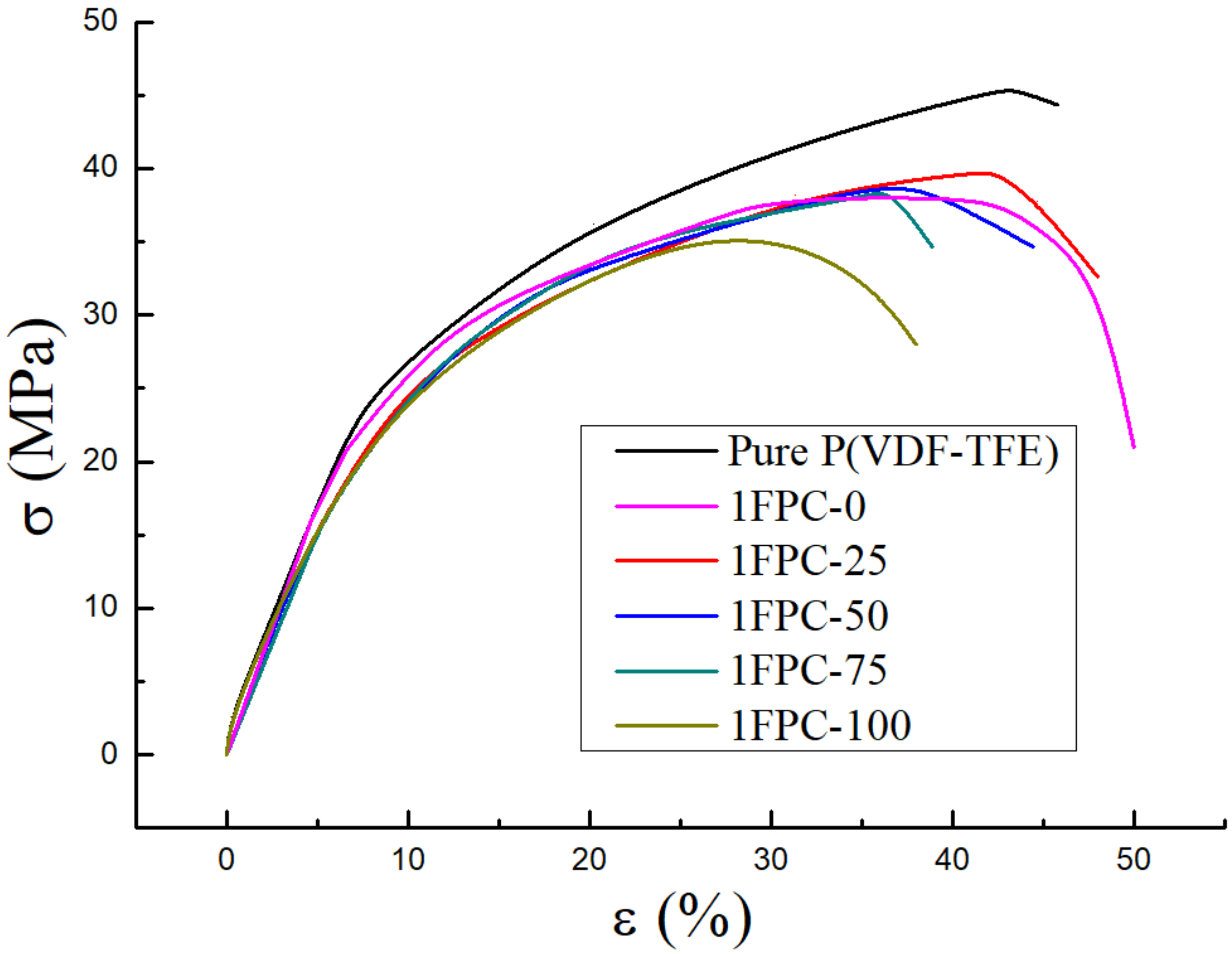

Figure S4. Typical compressive stress σ - strain ε curves for pure P(VDF-TFE) and composites filled by 1 % by weight of rGO, SWCNT or their mixtures.

Table S1. Main mechanical characteristics of the resulting composites.

Sample	Maximum deformation, %	Yield stress, MPa	Compressive strength, MPa	Elastic modulus, MPa
0.5FPC-0	36.4 ± 3.6	17.0 ± 0.1	35.9 ± 0.5	340.0 ± 0.5
0.5FPC-25	37.7 ± 4.8	22.0 ± 1.5	32.2 ± 2.4	255.9 ± 10.7
0.5FPC-50	40.1 ± 1.1	21.0 ± 2.8	34.7 ± 3.0	233.9 ± 7.4
0.5FPC-75	40.5 ± 12.8	19.8 ± 0.6	33.2 ± 0.7	233.1 ± 6.7
0.5FPC-100	43.6 ± 3.5	17.2 ± 0.2	43.1 ± 3.4	259.7 ± 4.9
1FPC-0	28.6 ± 0.9	20.0 ± 2.2	36.1 ± 0.5	392.2 ± 2.1
1FPC-25	37.1 ± 9.3	19.6 ± 0.4	43.1 ± 3.0	278.1 ± 6.0
1FPC-50	36.1 ± 0.6	18.8 ± 2.1	44.6 ± 3.7	321.1 ± 1.4
1FPC-75	38.1 ± 1.6	18.2 ± 0.3	45.9 ± 5.4	359.9 ± 4.1
1FPC-100	42.3 ± 3.6	16.9 ± 0.5	46.5 ± 2.9	378.7 ± 3.5
1.5FPC-0	26.3 ± 2.4	20.4 ± 0.5	34.2 ± 0.5	362.0 ± 0.5
1.5FPC-25	29.6 ± 0.9	21.6 ± 1.2	39.2 ± 0.6	253.0 ± 11.7
1.5FPC-50	36.7 ± 2.8	19.9 ± 0.1	37.6 ± 1.4	275.3 ± 3.5
1.5FPC-75	37.4 ± 6.8	21.4 ± 0.3	38.0 ± 0.7	281.3 ± 0.1
1.5FPC-100	43.0 ± 7.0	19.0 ± 0.2	43.2 ± 3.2	288.5 ± 6.3

Calculation of reflection, transmission and absorption coefficients

$$R = \frac{(n\text{-}1)^2 + k^2}{(n+1)^2 + k^2} \tag{S1}$$

$$T = (1 - R)^2 \exp(-ah); \tag{S2}$$

$$A = 1 - R - T; \tag{S3}$$

where *n* and *k* are the real and imaginary parts of the complex refractive index.

$$n = \sqrt{\frac{\varepsilon'}{2}\left(\sqrt{1 + \left(\frac{\varepsilon''}{\varepsilon'}\right)^2} + 1\right)}; \tag{S4}$$

$$k = \frac{\varepsilon''}{2n} \tag{S5}$$

$$a = \frac{4\pi k f}{c} \tag{S6}$$

where *c* is the speed of light, *f* is the frequency of the electromagnetic wave.
